# Visual Relationship Detection with Multimodal Fusion and Reasoning

**DOI:** 10.3390/s22207918

**Published:** 2022-10-18

**Authors:** Shouguan Xiao, Weiping Fu

**Affiliations:** 1 School of Mechanical and Precision Instrument Engineering, Xi’an University of Technology, Xi’an 710048, China; 2 School of Engineering, Xi’an International University, Xi’an 710077, China

**Keywords:** visual relationship detection, vision–language fusion, knowledge graph reasoning

## Abstract

Visual relationship detection aims to completely understand visual scenes and has recently received increasing attention. However, current methods only use the visual features of images to train the semantic network, which does not match human habits in which we know obvious features of scenes and infer covert states using common sense. Therefore, these methods cannot predict some hidden relationships of object-pairs from complex scenes. To address this problem, we propose unifying vision–language fusion and knowledge graph reasoning to combine visual feature embedding with external common sense knowledge to determine the visual relationships of objects. In addition, before training the relationship detection network, we devise an object–pair proposal module to solve the combination explosion problem. Extensive experiments show that our proposed method outperforms the state-of-the-art methods on the Visual Genome and Visual Relationship Detection datasets.

## 1. Introduction

With the rapid development of deep learning, computer vision has achieved good performance in many tasks, such as object classification [1,2], detection, and semantic segmentation. However, given an image, understanding the relationship between object-pairs is still a challenging task. It not only localizes the spatial and semantic information of object-pairs but also infers pairwise relationships. Visual relationships are usually expressed as triples <subject–predicate–object> [3,4,5]. They play an essential role in higher-level vision tasks, such as visual question answering [6], image captioning [7], and image generation [8]. There are many promising results in visual relationship detection works. For example, Qi et al. [9] proposed a method to caption better sports videos by modeling players’ interactions. Song et al. [10] devised a visual graph network to propagate semantic information to capture relationships.

Although the existing methods have achieved superior performance in relationship detection works, there are still two key dilemmas in this field, including combination explosion and non-exclusive label problems, as follows. (1) The combination explosion problem: prior works [11] follow the naive proposing method that if it extracts N objects from an image, there are N(N-1) object-pairs in the object-pair proposal state based on N detected objects. Even worse, multiple correlated relationships usually exist between two objects, and we tend to reserve more visual relationship triplets so that the combinations grow explosively. (2) The non-exclusive label problem. where, as different relationships in the label space have similar semantic information, a pair of objects may be associated with a group of predicates, not just one category. In particular, some predicates in the label space do not satisfy this assumption and have very similar semantic meanings, which results in blurred visual borders among these predicates. In other words, one visual object pair can be associated with a set of labels, not only a one-hot category [12].

To address these problems, there are two steps in our framework. First, we propose a relationship proposing module, which predicts whether a relationship exists between two objects. Relationship proposing filters out irrelevant pairings and only keeps relevant pairings. Moreover, it predicts the probability of the relationship and ranks scores. Second, we integrate two prediction modules. With the development of computer vision, especially the emergence of ViT [13], transformers have been applied to the multimodal research field. To capture useful semantic information, we propose a vision–language fusion module, which utilizes visual features and word embedding to predict the probability of the predicate. In addition, inspired by human cognition of relationships, we construct a knowledge graph reasoning module to further reveal predicate-level semantic correlations.

**Figure 1:**
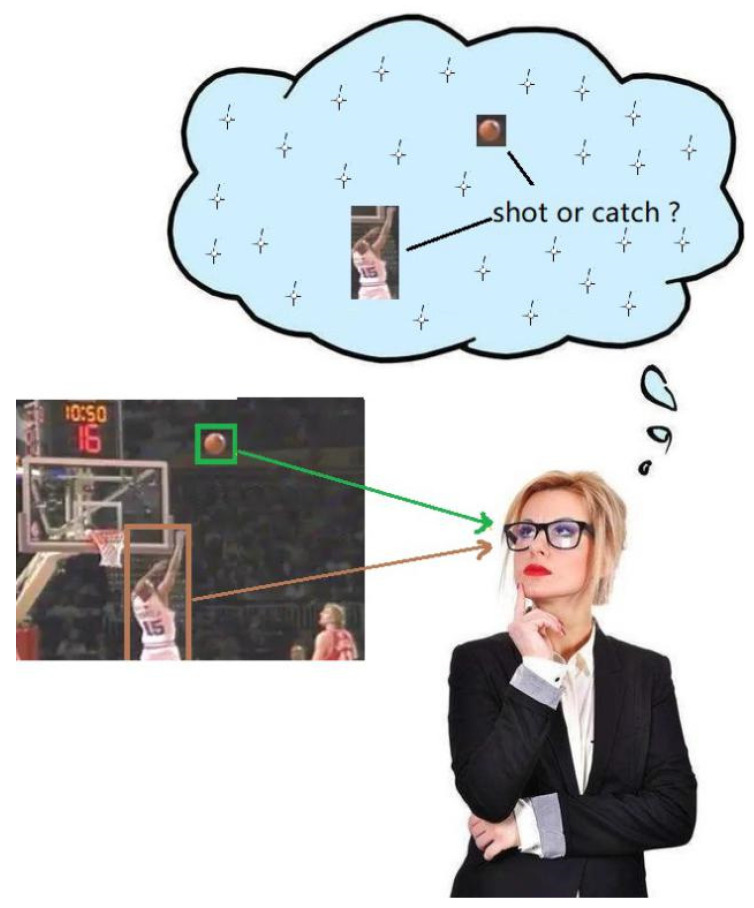
Person sees the scene and infers the relationship between the player and basketball.

As illustrated in Figure 1, when seeing the person and basketball, humans combine common knowledge with many factors to infer what relationship there is between the person and basketball. As a result, translation embedding is used to obtain the visual semantic relationship [7], and then the convolution neural network is used to integrate all the projection objects. The relationship features of the knowledge graph form a new relationship feature and encourage higher probabilities for all possible predicates. We summarize the contributions of our work below.

1. We propose a novel two-step prediction framework that unifies visual semantic relationship prediction, visual–language fusion prediction, and commonsense reasoning with a knowledge graph.
2. In the first step, we designed a relationship proposing module, which can effectively filter out irrelevant objects to solve the problem of combination explosion in visual relationship detection.
3. In the second step, we propose a relationship prediction model that fuses the two modules of visual–language fusion and knowledge graph reasoning. Visual–language fusion combines visual features and semantic embedding to find the potential association between objects. Knowledge graph reasoning integrates visual semantic relationships and external common knowledge to facilitate predicate inference.
4. Experiments on the Visual Genome and Visual Relationship Detection (VRD) datasets show that our proposed method performs better than current state-of-the-art methods, especially working well for infrequent relationships.

## 2. Related Work

In early works, visual relationship detection was developed as a phrase classification task [14] whose scale developed poorly since it was significantly dependent on sufficient training data [15]. Afterward, researchers proposed combining objects and predicates into triples for expressing relationships. For example, Lu et al. [16] first detected subjects and objects and then classified their predicates individually. Recently, as one of the most challenging problems in computer vision, visual relationship detection has been extensively investigated [17,18,19]. Li et al. [20] proposed a recurrent attention method, which can detect pipelines and focus on different parts of the image when given more than two predicates for object-pairs. Liu et al. [15] used the RGB-D information of images to represent inaccurate depth features for extracting semantic information. Qian et al. [6] propose to refine the scene graphs for improving the effectiveness and present a scene graph refinement network (SGR), which introduces a transformer-based refinement network to enhance the object and relation features for better classification. Wu et al. [21] propose to enhance video captioning with deep-level object relationships that are adaptively explored during training and present a transitive visual relationship detection (TVRD) module. They estimate the actions of the visual objects, and construct an object–action graph (OAG) to describe the shallow relationship between the objects and actions. Liu et al. [22] propose a multimodal similarity guided relationship interaction network (MSGRIN) to explicitly model the relations of relationships in graph neural network paradigm. The MSGRIN takes the visual relationships as nodes to construct an adaptive graph and enhances deep message passing by introducing entity appearance reconstruction, entity relevance filtering, and multimodal similarity attention.

Based on the above work, we propose a unified framework that is generally divided into two steps, which include object-pair proposal and predicate recognition [11]. First, the main task of object-pair proposal is to remove some unrelated object pairs. Researchers have performed some corresponding work in this regard. For example, Li et al. [23] scored a triplet non maximum suppression (NMS) to reduce the number of object-pairs. Zhou et al. [24] extracted the spatial relationship from an image and ranked the intersection over union (IOU) scores while ignoring the semantic relevance. Compared with previous methods, we integrate object spatial features, word semantic embedding, and attention mechanisms to derive relational predicates to predict object pair proposals and rank scores. Second, predicate recognition integrates vision–language fusion and graph reasoning with common sense knowledge [23,25]. Liu et al. [15] proposed integrating object features with the language prior and clustering-driven attention to infer visual predicates.

In our method, we make up for the above shortcomings and integrate the relationship between vision and language. Vision–language fusion takes object-mechanism feature information to analyze the existing characteristic relationship [25]. In addition, we also integrate the knowledge graph into the model so that it can correct the probability of a relational predicate by using common knowledge reasoning combined with the influence of the environment and improve the accuracy of relational expression.

## 3. Approach

In this section, the overall framework of our model is first introduced, and then we describe each module of the model in detail. Finally, the process of training and reasoning is shown.

**Figure 2:**
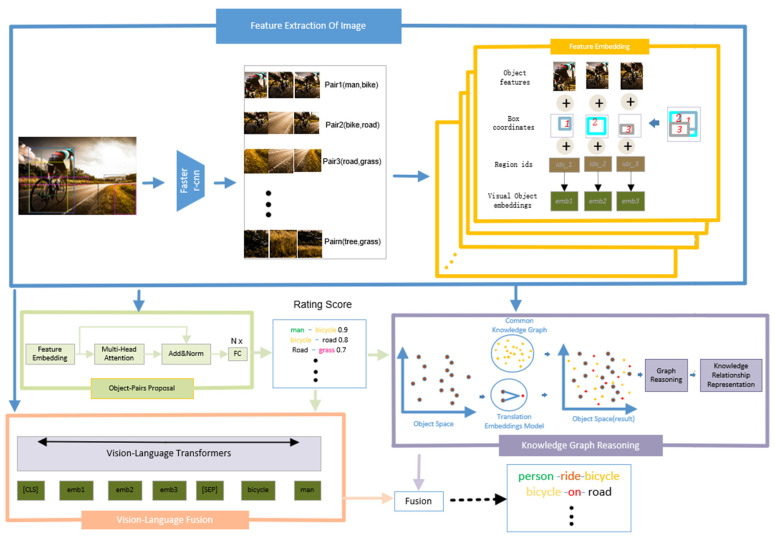
Structure of our proposed framework. A blue box is a module that is used to extract the features of an image and predict object labels. The green box can propose object-pairs and rate location embedding. The orange and brown boxes are the vision–language fusion module and knowledge graph reasoning module, respectively.

As illustrated in Figure 2, our framework is divided into four modules, including Feature Extraction of Image (blue box), Relationship Proposing (light green box), Vision-Language Fusion (pink box), Knowledge Graph Reasoning (brown box). Firstly, if receiving an image, Feature Extraction of Image is a pretrained detector that outputs a set of object labels, object features and corresponding bounding boxes. Then all of feature factors are fed into the Feature embedding module (yellow box) to output feature embedding. Feature embedding is fed into relationship proposing module to obtain a rating score of object proposal. Finally based on all of above factors, we utility vision–language fusion module and knowledge graph reasoning module to recognize predicates. 

### 3.1. Image Feature Extraction

In our work, gaining all of the information from the image is a crucial step for relationship detection. The following sections detail several components of image feature extraction.

We choose the pretrained object detection model Faster R-CNN [11] with the ResNet_101 backbone as the image detector. Given an image, it exactly detects all object labels $L=\{l_{i}\}$, object features $V={\left\{v_{i}\right\}}_{i\in N}$, and bounding boxes $B={\left\{x_{i},y_{i},w_{i},h_{i}\right\}}_{i\in N}$, where (*x_i_*, *y_i_*) is the upper left corner of *i*-th bounding box, *w_i_* is denoted as width and *h_i_* is height. The object-pairs are denoted as *N* (*N* − 1), and the corresponding feature of the union bounding box is:

(1) $${\mathrm{box}}_{\mathrm{union}}^{i,j}=\left[x_{i};y_{i};x_{j};y_{j};\ln\left(\frac{w_{i}}{w_{j}}\right);\ln\left(\frac{h_{i}}{h_{j}}\right);\ln\left(\frac{w_{j}}{w_{i}}\right);\ln\left(\frac{h_{j}}{h_{i}}\right)\right]$$

where $\ln\left(\frac{w_{i}}{w_{j}}\right)$ is the width ratio and $\ln\left(\frac{h_{i}}{h_{j}}\right)$ is the height ratio. To adjust it, we introduce a fully connected layer as follows:

(2) $$V_{\mathrm{emb}}={\left\{\mathrm{Relu}(\mathrm{Norm}(\mathrm{Linear}(V^{i})+\mathrm{Linear}(B^{i})+{\mathrm{idx}}^{i}))\right\}}_{i\in \{1,\;2,\;3\}}$$

As shown in Figure 2, the feature embedding module fuses the above information, and the region index for each region is denoted as $\mathrm{idx}={\left\{{\mathrm{idx}}^{i}\right\}}_{i\in \{1,\;2,\;3\}}$, calculating the embedding code of the image feature, denoted as:

(3) $$V_{\mathrm{emb}}={\left\{\mathrm{Relu}(\mathrm{Norm}(\mathrm{Linear}(V^{i})+\mathrm{Linear}(B^{i})+{\mathrm{idx}}^{i}))\right\}}_{i\in \{1,\;2,\;3\}}$$

where Linear(⋅) is denoted as full connection layer; Norm(⋅) denotes normalization function; and Relu(⋅) is denoted as nonlinear activation function.

### 3.2. Relationship Proposing

To determine whether two objects are related to one another, our proposed relationship proposal module is built on the feature extraction of the image module. The proposed relationship contains a multihead scaled dot-product attention sublayer [26], layer normalization [27], and *N* fully connected layers, where *N* is set to 3. The feature embedding of the feature extraction of an image module is denoted as $L_{i}\in R^{d_{w}},i\in K$, where *K* is the number of initial object-pairs. The calculation formula is represented as follows:

(4) $$X=\mathrm{softmax}(\frac{{\left(W_{0}^{}L_{i}^{}\right)}^{T}•(W_{1}^{}L_{i}^{})}{\sqrt{d_{w}/H}})$$

(5) $${\mathrm{head}}_{h}=(W_{2,h}L_{i}^{})X,h=1,\ldots ,H$$

(6) $${\tilde{L}}_{i}=W_{3}^{}[{\mathrm{head}}_{1},\ldots ,{\mathrm{head}}_{H}]$$

(7) $$D=\mathrm{LN}({\tilde{L}}_{i}+L_{i}^{})$$

where *H* is the number of attention heads. $W_{0}^{},W_{1}^{},W_{2}^{}\in R^{\frac{d_{w}}{H}\times d_{w}}$ are the projection matrices for the *H*-th head. LN(⋅) denotes layer normalization, *D* is input into fully connected layers, $Y=f_{3}(D)$, where $f_{3}(\cdot )$ is an output network implemented by three full connection layers. The probabilities of object-pairs being related to one another are denoted as *P*, defined as:

(8) $$P=\mathrm{softmax}(W_{4}Y)$$

To search the corresponding object pair closer to one human-annotated relationship in one image, we calculate the area of intersection between bounding-box objects detected by the detector and object annotations. In an image, relationship annotations are denoted as ${\left\{m_{<{\tilde{b}}_{\mathrm{sub}},{\tilde{b}}_{\mathrm{obj}}>}^{k}\right\}}_{k\in \{1,2,\ldots ,n\}}$, which describes the relationship feature between object-pairs. The target combination that should be obtained is represented as $M_{<{\tilde{b}}_{\mathrm{sub}},b_{\mathrm{obj}}>}$. The overlap rate between object-pairs is defined as:

(9) $$p=\max(\{\mathrm{IOU}(m_{<{\tilde{b}}_{s\mathrm{ub}},b_{\mathrm{sub}}>}^{k})\cdot \mathrm{IOU}(m_{<{\tilde{b}}_{\mathrm{obj}},b_{\mathrm{obj}}>}^{k}{)\}}_{k\in \{1,2,\ldots ,n\}})$$

where IOU(⋅) is denoted as the ratio of intersection and union of two bounding-boxes. The larger *p* is the closer that the corresponding object-pair is to one human-annotated relationship.

We make the binary rating label λ indicate whether a relationship exists between object-pairs and set λ as 1 or 0, as follows:

(10) $$\lambda =\left\{ \begin{array}{l} 1\;\;\;\;\;p>0.5\\ 0\;\;\;\;\;p<0.3 \end{array}\right.$$

Object-pairs are removed when *p* is between 0.3 and 0.5 in the training process. The loss is:

(11) $$L_{\mathrm{pr}}=\frac{1}{K}\sum_{k=1}^{K}\left[\lambda_{k}\log P_{k}+(1-\lambda_{k})\log(1-P_{k})\right]$$

where *K* is the batch size.

### 3.3. Proposal Scores of Object-Pairs

Plausible proposals are produced from the outputs of the pretrained object-pair proposal model. We calculate the probability of each object-pair $<o_{i},o_{j}>$, and the proposal score is defined as

(12) $$P_{r}^{ij}=P_{<o_{i},o_{j}>}\cdot \Phi (o_{i})\cdot \Phi (o_{j})$$

where Φ(⋅) is the probability of an object from the object detector. The rating score comes from the ranking proposal of object-pairs by proposal scores $P_{r}^{ij}$.

### 3.4. Vision–Language Fusion

Based on the output of the feature extraction of an image module, we encode feature embedding and object labels as inputs and use bidirectional multimodal transformers [12] as backbones. As shown in Figure 2, vision–language fusion, text embedding, and visual features are concatenated as input, represented by $t=\{t_{1}^{v},\ldots ,t_{\left|v\right|}^{v},t_{1}^{x},\ldots ,t_{n}^{x}\}=Cod(e^{v},e^{x})$. As discussed in Section 3.1, the feature extraction of an image module attains the feature vector denoted as $e^{v}=\{{\mathrm{emb}}_{1},{\mathrm{emb}}_{2},{\mathrm{emb}}_{3}\}$ and $e^{x}=\{\mathrm{encode}({\mathrm{label}}_{1}),\mathrm{encode}({\mathrm{label}}_{2})\}$. The multimodal transformers architecture consists of an encoder and decoder. The encoder is a stack of m transformer encoder blocks denoted as $E_{m}$, and the decoder is a stack of m transformer decoder blocks denoted as $D_{m}$. Each transformer encoder block consists of a self-attention layer and a fully connected layer with additional residual connections [2]. However, the transformer decoder has an additional cross-attention layer compared with the encoder in each block. The output of the encoder is $o_{e}=E_{m}(t)$ and the output of the decoder is $o_{d}=D_{m}(o_{e},y_{i})$, where $y_{i}$ denotes the decoder’s input token. Finally, the probability of feature text tokens is predicted.

(13) $$P_{\theta }(y_{i+1}|y_{i},t)=\frac{\exp o_{d}{}^{i+1}}{\sum_{y_{j}\in Y}\exp o_{d}{}^{y_{j}}}$$

The model parameters θ are trained by minimizing the negative log-likelihood of text embedding and visual feature vector as:

(14) $$L_{\mathrm{vlf}}=L_{\mathrm{cel}}(P_{\mathrm{pred},n},y_{n})=-\sum_{i=0}^{\left|y\right|}\log P_{\theta }(y_{i+1}|y_{i},t)$$

where the initial input token $y_{0}$ is a start-of-sequence token and $L_{\mathrm{cel}}$ is the cross-entropy loss.

### 3.5. Knowledge Graph Reasoning

In Figure 2, for knowledge graph reasoning, the object space contains the entities obtained from the image and represented by the brown color. At the same time, we map relationships from a commonsense knowledge graph to the object space, represented by yellow. In the knowledge graph, the relationship between entities is expressed by translation embedding models, subject+predicte≈object. Moreover, it can also solve the relationship characteristics of visual objects. From Sec. A, we can obtain all the object features, which are denoted as $V^{D^{d}}$, $v_{i},v_{j}\in V^{D^{d}}$, where $v_{i}$ is the object and $v_{j}$ is the subject. The relationship formula between two entities can be expressed as follows:

(15) $$W_{\mathrm{fs}}v_{i}+r_{\mathrm{so}}\approx W_{\mathrm{fo}}\;v_{j}$$

where embedding matrices $W_{\mathrm{fs}},W_{\mathrm{fo}}\in R^{u\times D^{d}}$ and relationship vector $r_{\mathrm{so}}\in R^{u}$.

Based on feature extraction of the image, the spatial union box feature $v_{ij}$ between two objects can be obtained. The relationship representation can be deduced $R_{ij}$ from Formula (15):

(16) $$R_{\mathrm{ij}}=(W_{\mathrm{fo}}v_{j}-W_{\mathrm{fs}}v_{i})\circ v_{\mathrm{ij}}$$

where $R_{\mathrm{ij}}\in R^{u\times D^{d}}$ is a relationship group indicating k relationships between object-pairs.

To select the correct relationship predicate, it distinguishes the relationship between two objects. Transformation matrices $W_{\mathrm{vl}}\in R^{(D^{d}+L)\times D^{c}}$ map $W_{l}(l_{i},l_{j})\in R^{u\times L}$ and $R_{\mathrm{ij}}$ concatenation matrix, resulting in multiple groups of entities’ evolved features, as following:

(17) $$R_{a}=\sum_{l_{i},l_{j}\in L}W_{\mathrm{vr}}[R_{\mathrm{ij}},W_{l}(l_{i},l_{j})]W_{\mathrm{vl}}$$

where $R_{a}\in R^{D^{c}\times D^{c}}$, $W_{vr}\in R^{D^{c}\times u}$ is a transformation matrix.

As human common knowledge has the function of reasoning about the relationship between two entities, we will use common knowledge semantics to constrain the representation of nodes in the knowledge graph. $\hat{A}$ is an adjacency matrix that contains the original *A* and identity matrix *I* and is denoted as $\hat{A}=A+I$ in graph G. We use graph convolutional networks [28] such that all rows sum to one, i.e., $Q^{-\frac{1}{2}}AQ^{-\frac{1}{2}}$, where Q is the diagonal node degree matrix of *A*. Relational knowledge presentation is $S_{\mathrm{mc}}\in R^{M\times D^{c}}$ and the formulation is represented as:

(18) $$S_{\mathrm{mc}}=\sigma ({\hat{Q}}^{-\frac{1}{2}}\hat{A}{\hat{Q}}^{-\frac{1}{2}}[R_{a},S]W_{m})$$

where $S\in R^{D^{c}\times L}$ maps the common relationship from the knowledge graph.

For making $S_{\mathrm{mc}}$ refine relationship characteristics, we extended the concatenation of R and $S_{\mathrm{mc}}$, representing $\hat{R}\in R^{N\times M\times D^{d}}$ and ${\hat{S}}_{mc}\in R^{N\times M\times D^{c}}$. Through a trainable weight matrix, $W_{h}\in R^{(D^{d}+D^{c})\times 1}$ evaluates the compatibility of relational knowledge presentation, resulting in $S_{rt}$

(19) $$S_{\mathrm{rt}}=\sigma (\left\lfloor \hat{R},{\hat{S}}_{\mathrm{mc}}\right\rfloor W_{h})S_{\mathrm{mc}}W_{\mathrm{st}}$$

where $W_{\mathrm{st}}\in R^{D^{c}\times D^{d}}$ is a transformation matrix, adjusting the dimension of output. $S_{\mathrm{rt}}\in R^{N\times D^{d}}$ is N groups of relational knowledge presentations.

To enhance relationship representations, we fuse the spatial relationship $R_{\mathrm{ij}}$ and the common knowledge relationship $s_{\mathrm{rt}}^{\mathrm{ij}}\in S_{\mathrm{rt}}$ is as follows:

(20) $$P_{\mathrm{ij}}{}^{r}=\mathrm{softmax}(\alpha W_{r}^{p}R_{\mathrm{ij}}+\beta W_{s}^{p}s_{\mathrm{rt}}^{\mathrm{ij}})$$

where α and β are the trade-off parameters and $W_{r}^{p}$ and $W_{s}^{p}$ are learned parameters.

### 3.6. Training and Inference Procedures

During the training stage, we unify vision–language fusion and knowledge graph reasoning into the overall framework, and the training loss is defined as:

(21) $$L_{r}=\alpha L_{\mathrm{vlf}}+\beta L_{\mathrm{cel}}(P_{\mathrm{ij}}^{r},y_{n})$$

## 4. Experiments

In the following work, we perform experiments to verify the proposed model, compare it with current state-of-the-art methods and use charts to show the relevant detection results. In addition, we also adjust the hyper-parameters and configuration structure of the model and then analyze their impact on the results.

### 4.1. Datasets

Visual Relationship Dataset (VRD) [11]. VRD is a previous dataset using relational triples as annotations for visual relationship detection. It consists of 70 predicate categories, 100 object categories and 5000 images. We use 20% of the images for testing and 80% of the images for training. In addition, there are 6672 unique relationships and 37,993 relationship instances.

Visual Genome (VG) [29]. VG is a larger scale relationship dataset than VRD. Currently, the pruned version of VG contains 19,237 unique relations, 1,174,692 relation instances, 200 object categories, and 100 predicate categories. In addition, there are 99,658 images, consisting of 73,801 images for training and 25,857 for testing [30].

### 4.2. Analysis of Common Sense Knowledge

We extract structured information such as entities, relationships, and entity attributes from semi-structured and unstructured data. After acquiring new knowledge, they are integrated to eliminate contradictions and ambiguities, for some entities may have multiple expressions, and a specific title may correspond to multiple different entities. As the knowledge obtained by the automatic extraction method often has a large number of missing relationships, we further complete the knowledge based on the existing knowledge. For the new knowledge that has been merged, the qualified part can be added to the knowledge base only after the quality evaluation (part of which needs to be manually screened) to ensure the quality of the knowledge graph. In knowledge graphs, there are 219,506 relationship instances, 816 object categories, and 113 predicate categories.

### 4.3. Evaluation of Model

Phrase detection. Given an image, phrase detection aims to indicate what the relation is between objects and output the triplet of labels <subject–predicate–object>. There are correct labels and bounding box proposed has more than 0.5 IOU with the ground truth box.

Relationship detection. Relationship detection not only detects two correct object labels and corresponding bounding boxes, but also it locates their IOU with the ground truths more than 50% of which each box has. Equally, the output is a relationship triplet of labels <subject–predicate–object>.

Predicate Classification. With the ground truth boxes and categories of object given, the task of predicate classification is to predict possible predicates between the objects.

### 4.4. Experimental Environment and Parameter Settings

In the experiment, our server is conducted on a single NVIDIA Quadro RTX 8000 GPU and 128 G RAM. We implement our method with the PyTorch [11] framework and use Faster R-CNN as the object detector. Adam [31] is used as the optimizer with an initial learning rate of 0.00001. We trained our model for 60 epochs on the VRD and on the VG.

### 4.5. Comparison with the State-of-the-Art Methods

BLOCK [17] combines image features and semantic embedding, ignoring spatial location information between the subject and object. Compared with BLOCK, Zoom-Net [32] performs better, fusing image features, spatial location information, and semantic embedding. Based on fusing the three features mentioned above, HGAT [29] constructs an object-level attention graph and a triplet-level attention graph, and MF-URLN [33] explores undetermined relationships and achieves significant improvements. In contrast to MF-URLN, which directly incorporates determinate confidences into final predictions, TCE [11] utilizes rating scores that indicate probabilities of objects being related to one another to select plausible proposals to reduce computational complexity. Inspired by the previous methods, we integrate all the advantages of the previous methods and achieve better performance.

To prove the advantages of our method, we compare our model with the above state-of-the-art methods and describe the detailed results of relation detection, phrase detection, and predicate detection in the following sections.

#### 4.5.1. Experiments on the VRD

**Figure 3:**
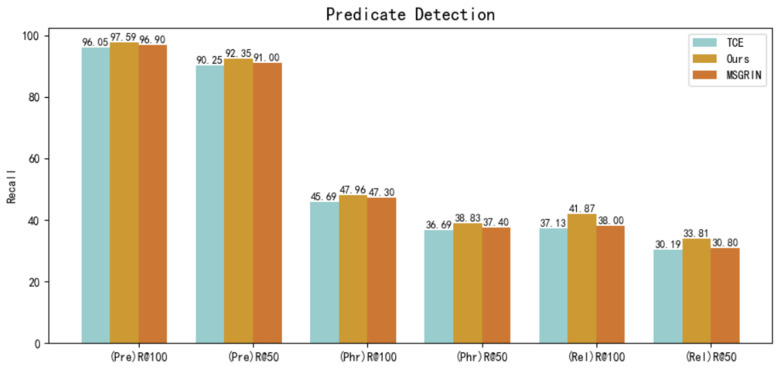
While k is set 70, our method surpasses TCE and MSGRIN in R@n, reaching state-of-the-art results in predicate detection.

We compare our method with the representative method TCE and MSGRIN [22] at present in R@n; when n is set to a different parameter, the result is greatly impacted. As shown in the Figure 3, our method considerably improves and our model outperforms TCE, e.g., 92.35% vs. 90.25% for R@50 in predicate detection. In different tasks, the performance of the model is also different. In relationship prediction, our model outperforms MSGRIN by 3% and 41.87% vs. 38% for R@100, while our model outperforms MSGRIN 33.81% vs. 30.8% for R@50. Compared with TCE and MSGRIN, we have added attention mechanism in our model to associate image features with relationships, which produces better results.

Based on R@n, we introduce the top-k predicate after sorting the confidences of predicates between a pair of objects. Hyper-parameter k is set to 1 and 70 in per n value. As illustrated in Table 1, compared to TCE, our model achieves comparable performances in predicate detection, especially 61.13% vs. 57.93% for R@50, k = 1. In addition, our method outperforms MSGRIN by more than 2% on all sets in the relation detection.

Since the number of training instances is even smaller than the possible triplet combinations, it is important to detect unknown relationships for the model. In a real environment, the relational semantics are similar between two different object-pairs. For example, <person-sit on-desk> and <person-sit on-chair>. A successful model should have the ability to generalize similar predicates. We evaluate our model in zero-shot detection and compare it with current state-of-the-art methods, the results are listed in Table 2. As we used the common sense relationship in the knowledge graph to reason the relationship of object-pairs in the image, our model performs better than other methods in all sets.

#### 4.5.2. Experiments on the VG

To further validate that our method outperforms other methods, we evaluated our method with the different metrics on VG. 

As illustrated in the Table 3, our model performs better than the other methods in all sets. We compare the method with TCE in R@n, k = 1, and our method respectively yields 3.37% and 4.14% gains for R@50 and R@100 in relationship detection. In addition, in R@n, k = 100, our model significantly outperforms MSGRIN (e.g., for R@100, the result increases from 23.19% to 26.95% and for R@50, it increases from 19.51% to 21.82%).

In summary, in the process of semantic understanding, TCE uses a fully-connected network, while we use bi-transformers [12], which include an attention module and a fully-connected network. Thus, by capturing the semantic information, our method performs better than TCE. Moreover, we introduce a knowledge graph for common knowledge reasoning, which not only helps to enhance the accuracy of relational predicates but can also greatly promote the prediction of similar predicates in zero-shot. In the next section, we compare the result of the method without common knowledge reasoning with the complete model from the perspective of visualization.

#### 4.5.3. Qualitative Comparison of Our Model

**Figure 4:**
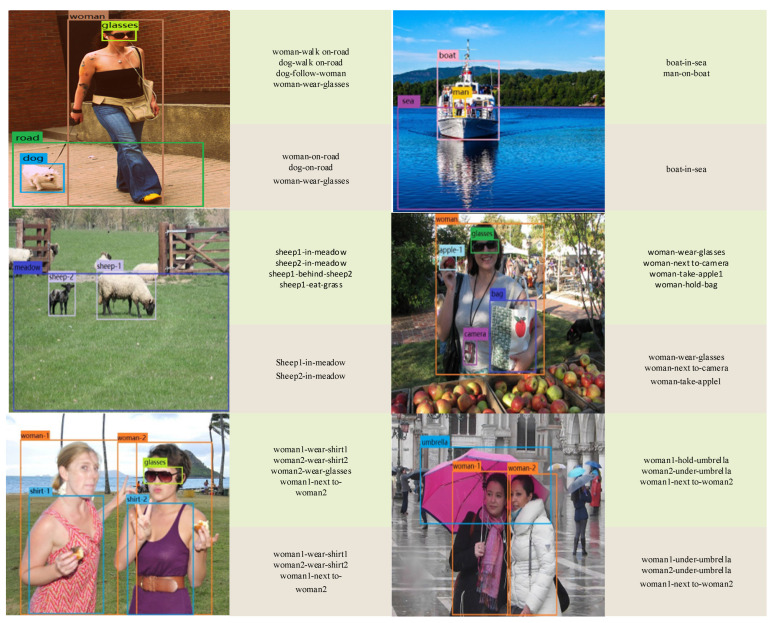
Qualitative results of our model on the VRD. On the right of each image, the green box shows the positive prediction in the top 50 predictions of our full model. The brown box shows that of our model without common knowledge reasoning.

To verify the effect of common knowledge reasoning, we compare our full method and the method without the knowledge graph reasoning module, listing their results in the Figure 4. As the knowledge graph includes all of environmental factors, the accuracy of relationship detection between objects improves significantly and more relationships are deduced in line with common knowledge. Moreover, the predicate between two objects will be reasonably adjusted. In particular, it achieves a quite good effect on detecting the predicate with similar semantics in zero shots. 

## 5. Conclusions and Future Work

In this paper, we devise a unified network architecture for visual relationship detection, which is the supervised model to realize triplet <subject–predicate–object>. We first propose an object-pair proposal module to predict plausible proposals. Second, we fuse the vision–language fusion module and the knowledge graph reasoning module to capture features of relationships from different perspectives, including attention-level information and knowledge graph reasoning. In particular, we inject external common sense knowledge to support the visual common sense reasoning task, which greatly promotes the accuracy of predicate-detection. The experiment on the VRD and VG datasets shows that our method outperforms the state-of-the-art methods. 

In future work, we will attempt to extend our method to operate mobile robots, hoping that relationship detection can play a great role in visual language navigation in the real world.

## Figures and Tables

**Table 1:** Comparison with previous methods on VRD, where bold font indicates the best results.

Method	Predicate Detection			Phrase Detection				Relationship Detection			
	R@100/50	R@100	R@50	R@100	R@50	R@100	R@50	R@100	R@50	R@100	R@50
	k = 1	k = 70	k = 70	k = 1	k = 1	k = 70	k = 70	k = 1	k = 1	k = 70	k = 70
Zoom-Net [32]	55.98	94.56	89.03	28.09	24.82	37.34	29.05	21.41	18.92	27.30	21.37
BLOCK [17]	-	92.58	86.58	-	-	28.96	26.32	-	-	20.96	19.06
TCE [11]	57.93	96.05	90.25	40.01	33.46	45.69	36.69	31.37	26.76	37.13	30.19
MF-URLN [33]	58.20	-	-	36.10	31.05	-	-	26.80	23.90	-	-
HGAT [29]	59.54	97.02	90.91	-	-	-	-	24.63	22.52	27.73	22.90
MSGRIN [22]	57.9	96.9	91.0	-	33.8	47.3	37.4	-	27.2	38.0	30.8
Ours	**61.13**	**97.59**	**92.35**	**42.55**	**35.71**	**47.96**	**38.83**	**34.43**	**29.95**	**41.87**	**33.81**

**Table 2:** Comparison with previous methods on the VRD zero-shot set; bold font indicates the best results.

Method	Predicate Detection			
	k = 1		k = 70	
	R@100/50	R@50	R@100	R@50
MCN [34]	26.7	26.7	-	-
TCE	26.52	26.52	86.66	72.97
MSGRIN	-	-	89.15	75.28
Ours	**29.13**	**29.13**	**89.87**	**75.95**

**Table 3:** Comparison with state-of-the-art methods on VG; bold font indicates the best results.

k	Methods	Predicate Detection		Phrase Detection		Relationship Detection	
		R@100	R@50	R@100	R@50	R@100	R@50
1	MF-URLN	72.20	71.90	32.10	26.60	16.50	14.40
	TCE	71.25	70.95	34.31	26.90	21.45	17.22
	MSGRIN	71.64	71.23	33.49	26.35	21.08	16.79
	Ours	**73.32**	**72.69**	**36.83**	**28.76**	**25.59**	**20.59**
100	TCE	96.23	91.19	35.04	27.75	22.82	18.47
	MSGRIN	96.58	91.36	35.47	27.82	23.19	19.51
	Ours	**97.86**	**92.79**	**36.92**	**28.66**	**26.95**	**21.82**

## Data Availability

Data available on request.
